# A non-canonical promoter element drives spurious transcription of horizontally acquired bacterial genes

**DOI:** 10.1093/nar/gkaa244

**Published:** 2020-04-16

**Authors:** Emily A Warman, Shivani S Singh, Alicia G Gubieda, David C Grainger

**Affiliations:** Institute of Microbiology and Infection, School of Biosciences, University of Birmingham, Edgbaston, Birmingham B15 2TT, UK

## Abstract

RNA polymerases initiate transcription at DNA sequences called promoters. In bacteria, the best conserved promoter feature is the AT-rich -10 element; a sequence essential for DNA unwinding. Further elements, and gene regulatory proteins, are needed to recruit RNA polymerase to the -10 sequence. Hence, -10 elements cannot function in isolation. Many horizontally acquired genes also have a high AT-content. Consequently, sequences that resemble the -10 element occur frequently. As a result, foreign genes are predisposed to spurious transcription. However, it is not clear how RNA polymerase initially recognizes such sequences. Here, we identify a non-canonical promoter element that plays a key role. The sequence, itself a short AT-tract, resides 5 base pairs upstream of otherwise cryptic -10 elements. The AT-tract alters DNA conformation and enhances contacts between the DNA backbone and RNA polymerase.

## INTRODUCTION

All living organisms transcribe their genomes using the enzyme RNA polymerase (1). The process initiates at defined DNA sequences called promoters (1). In *Escherichia coli*, a multisubunit core RNA polymerase (α_2_ββ') binds one of seven dissociable σ factors to recognise promoter DNA (2). The housekeeping σ^70^ factor is best studied and targets two promoter regions; the -10 (5′-TATAAT-3′) and -35 elements (5′-TTGACA-3′) (2). The -10 sequence facilitates promoter DNA unwinding and is usually indispensable (3). Conversely, the -35 element aids initial RNA polymerase recruitment and can be replaced by transcription factors fulfilling the same role (3). In isolation, core promoter elements are ineffective (4). For instance, specific interactions between σ^70^ and the -10 element occur only in the context of single stranded DNA (5). Hence, -10 elements support DNA melting but not sequence specific RNA polymerase recruitment (4,5).

Sections of the *E. coli* genome acquired by horizontal gene transfer have an unusually high AT-content (6). Consequently, sequences resembling promoter -10 elements occur frequently (7). These can participate in spurious transcription initiation (8–10). The histone-like nucleoid structuring (H-NS) protein counteracts this by coating large AT-rich DNA islands (8,11,12). The resulting nucleoprotein complexes hinder transcription (13,14). Hence, H-NS acts as a xenogeneic silencer (15,16). Importantly, silencing prevents titration of RNA polymerase that otherwise perturbs housekeeping transcription (8). Given the lack of ancillary promoter elements, and binding sites for transcription factors, it is not clear how RNA polymerase initially recognizes promoters within horizontally acquired genes.

In this paper, we sought to understand recognition of spurious promoters by RNA polymerase. Our study reveals the importance of a short AT-tract, positioned upstream of the -10 element, at happenstance but not conventional promoters. This unusual sequence element permits transcription from otherwise cryptic -10 hexamers. Mechanistically, the AT-tract facilitates an interaction with σ^70^ residue R451, which recognizes the DNA backbone (17). We show that uncontrolled transcription of AT-rich genes is not restricted to σ^70^ dependence; the alternative σ^38^ factor also plays a role.

## MATERIALS AND METHODS

### Strains, plasmids and oligonucleotides

Strains, plasmids and oligonucleotides are described in Table 1. Standard procedures for cloning and DNA manipulation were used throughout. Promoter DNA fragments were made either using olignonucleotides or synthetic DNA fragments described in Table 1. All promoter DNA fragments were flanked by EcoRI and HindIII restriction sites to allow cloning in plasmid pRW50 or pSR. To construct DNA fragments with random sequence, but defined AT-content, we used the oligonucleotide ‘Random R’ in combination with variants of the ‘Random F’ primer (Table 1). Each variant of the latter was synthesised using a different mixture of nucleotides to generate ‘any base’ (N). Whilst the A:T and G:C ratios were always the same the overall AT-content varied as indicated. Primers used to generate other synthetic promoter sequences, with or without AT-tracts, are also listed in Table 1. These promoters were made using pairs of oligonucleotides with short regions of complementarity at the 3′ end with the remainder of the sequence serving as a template for DNA polymerase. The AT^R^ fragment introduced a random string of A or T bases (denoted W in Table 1). RPB104 *Δhns* was constructed by Gene Doctoring as described previously (8,18).

**Table 1.** Strains, plasmids and oligonucleotides

Name	Description^a,b,c^	Source
	*Strains*	
JCB387	Δ*nir* Δ*lac*	(33)
RPB104 *Δhns*	RPB104MG1655 with C-terminal SPA-tagged *rpoS*	(23)
	RPB104 *hns::kan*	This work
	*Plasmids*	
pRW50	low copy number *lac* fusion vector with *Eco*RI/*Hin*dIII cloning site and Tet^R^	(34)
pSR	pBR322-derived vector with *Eco*RI/*Hin*dII cloning site upstream of λ*oop* terminator.	(20)
	Encodes Amp^R^.	
pSR Δ45-9A-10T	pSR carrying an optimised derivative of the *cbpA* promoter	(17)
	*Primers for generating random DNA sequences*	
Random F	GGCTGCGAATTCNNNNNNNNNNNNNNNNNNNNNNNNNNNNNNNNNNNNNN	This work
	NNNNNAGGAGGATGCGGACTATG	
Random R	CGCCCGAAGCTTcatagtccgcatcctcct	This work
	*Primers for generating synthetic promoter sequences*	
-10 F	GGCTGCGAATTCgaccggcgagcttcgcagtcagctgac**tataat**tgccgcgcgca	This work
-10 R	CGCCCGAAGCTTcatagtccgcatcctcctgcgcgcggcaattatagtcagctgac	This work
-10/-35TT F	GGCTGCGAATTCgacc**tt**cgagcttcgcagtcagctgac**tataat**tgccgcgcgca	This work
-10/AT^i^ F	GGCTGCGAATTCgaccggcgagcttcgc**tatttat**tgac**tataat**tgccgcgcgca	This work
-10/AT^i^ R	CGCCCGAAGCTTcatagtccgcatcctcctgcgcgcggcaattatagtcaataaat	This work
-10/AT^i^/-35TT F	GGCTGCGAATTCgacc**tt**cgagcttcgc**tatttat**tgac**tataat**tgccgcgcgca	This work
-10/AT^ii^ F	GGCTGCGAATTCgaccggcgagcttcgcag**aattt**tgac**tataat**tgccgcgcgca	This work
-10/AT^ii^ R	CGCCCGAAGCTTcatagtccgcatcctcctgcgcgcggcaattatagtcagctgac	This work
-10/AT^ii^/-35TT F	GGCTGCGAATTCgacc**tt**cgagcttcgcag**aattt**tgac**tataat**tgccgcgcgca	This work
-10 general R	GCCCGAAGCTTCatagtccgcatcctcctgcgcgcggcaattatagtc	This work
	*Primers for introducing an AT-rich spacer of random sequence*	
-10/AT^R^ F	GGCTGCGAATTCgaccggcgagcttcgcwwwwwwwtgactataattgccgcgcgc	This work
-10/AT^R^/-35TT F	GGCTGCGAATTCgacc**tt**cgagcttcgcwwwwwwwtgactataattgccgcgcgc	This work
	*Primers for amplifying intragenic promoters*	
*wzxB* 1.1 F	GGCTGCGAATTCacgttactttatctttactatctgc	This work
*wzxB* 1.1 R	GCCCGAAGCTTCCTCCTttgtaagaacacttggtcctgaaaa	This work
*yigG* 1.2 F	GGCTGCGAATTCtactccattatctcgtcatcaacatg	This work
*yigG* 1.2 R	GCCCGAAGCTTCCTCCTcattgcctgaacaggcaaaatcttc	This work
*yqiI* 2.2 F	GGCTGCGAATTCataagttacaccgaaagtataagag	This work
*yqiI* 2.2 R	GCCCGAAGCTTCCTCCTgaatattttatgaatgttttctg	This work
*ygaQ* 1.1 F	GGCTGCGAATTCcggttacacaatactaacttatttaac	This work
*ygaQ* 1.1 R	GCCCGAAGCTTCCTCCTtgaaaaatcaatggcgcttaaatcatc	This work
*wcaD* F	GGCTGCGAATTCTcaaacagtttggtatcaaaacg	This work
*wcaD* R	GCCCGAAGCTTCATAGTCCGCATCCTCCTcccctgaaaacgatccgg	This work
*lpxD* F	GGCTGCGAATTCAccagtgccagattgcacataacg	This work
*lpxD* R	GCCCGAAGCTTCATAGTCCGCATCCTCCTtcaggctgcccgccataatgacg	This work
	*Overlapping primers replacing AT-rich spacers with a GC-rich spacer*	
*wcaD* GC fwd	GGCTGCGAATTCtcaaacagtttggtatcaaacttcgcagtcag**cttgctatgat**	This work
*wcaD* GC rev	AGCCCGAAGCTTcctcctcccctgaaaacgatccggataatattatccctgcgagaat	This work
	catagca**agctgactgcgaag**tttgat	This work
*lpxD* GC fwd	GGCTGCGAATTCaccagtgccagattgcac**cttcgcagtcagctgac**gacaat	This work
*lpxD* GC rev	AGCCCGAAGCTTcctccttcaggctgcccgccataatgacgccaccggcaaccgccgt	This work
	attgtcgtcagctgactgcgaaggtgcaa	This work
	*Synthetic DNA fragments to replace AT-rich spacers with GC-rich spacer*	
*wzxB*1.1 gc_Spacer	CTTGAGTCCACGCTAGATCTGGCTGCGAATTCAcgttactttatctttactatctgctg	This work
	ctttggcaatactctgagttgctgtgagattgaaa**cttcgcagtcagctgac**tatcatatatagcatagtcg	
	cttggcaaaaaccgaatataccgaaattttcaggaccaagtgttcttacaaaggaggAAGCTTCGG	
	GCTTGTCAGTGCGCAAAAAGAT	
*yigG*1.2 gc_spacer	CTTGAGTCCACGCTAGATCTGGCTGCGAATTctactccattatctcgtcatcaacatga	This work
	attgccagcgactccgtgatagtggtttcatctatata**cttcgcagtcagct**tggtacattagcagtatatatc	
	atctctatcatcacaatgatagccgaagattttgcctgttcaggcaatgaggaggAAGCTTCGGGCTT	
	GTCAGTGCGCAAAAAGAT	
*yqiI*2.2 gc_spacer	CTTGAGTCCACGCTAGATCTGGCTGCGAATTCataagttacaccgaaagtataagagtt	This work
	ttgattataaaagtcttgacct**cttcgcagtcagctgac**tatatttgcccatgcagatgggtattcttctcctggag	
	atgggcctggtagtgcattattacagaaaacattcataaaatattcaggaggAAGCTTCGGGCTTGT	
	CAGTGCGCAAAAAGAT	
*ygaQ*1.1 gc_spacer	CTTGAGTCCACGCTAGATCTGGCTGCGAATTCcggttacacaatactaacttatttaac	This work
	ccaaaatatcataaaaaagccgttatgaat**ttcgcagtcagct**tggtaacttgtcagttggatgaacaacaa	
	atgtcatcactgctttatgaaagagatgatttaagcgccattgatttttcaaggaggAAGCTTCGGGCTT	
	GTCAGTGCGCAAAAAGAT	

^a^N is either A, C, G or T incorporated into the oligonucleotide at random but supplied at a defined % of each nucleotide. Used to generate the DNA fragment library described in Figure 1.

^b^W is A or T, with an equal likelihood of either base being incorporated.

^c^Synthetic promoter -10 elements are underlined and key base changes introduced by oligonucleotides are in bold.

### β-Galactosidase assays

Assays were done following the Miller protocol using *E. coli* strain JCB387 (19). Cells were grown to mid-log phase in LB media, supplemented with 35 μg/ml tetracycline, at 37°C. All experiments, except for the AT^R^ assays, were done in triplicate and mean values are shown. For the AT^R^ assays we present the overall distribution of activities obtained from single experiments. Error bars represent standard deviation.

### Proteins

RNA polymerase core enzyme was purchased from NEB. WT and R451A σ factors were purified as previously described (17).

### *In vitro* transcription

*In vitro* transcription assays were done using the system of Kolb *et al.* (20) and the protocol of Savery *et al.* (21). Briefly, pSR carrying promoters of interest was isolated using a QIAGEN maxiprep kit. Plasmid DNA was mixed to a final concentration of 16 μg/ml with transcription buffer (20 mM Tris pH 7.9, 200 mM GTP/ATP/CTP, 10 mM UTP, 5 μCi (α32P) UTP, 5 mM MgCl_2_ and 100 μg/ml BSA). RNA polymerase was mixed with either WT or R451A σ^70^ then added to reactions for 10 min at 37°C. RNA products were visualized on a 7% denaturing polyacrylamide gel. RNAI transcript was used as a loading control. Full gel images are shown in Supplementary Figure S1.

### Promoter DNA bending assays

To compare differences in DNA bending, double stranded promoter fragments generated by PCR were separated on a 7.5% non-denaturing polyacrylamide gel. Electrophoresis was done in TBE buffer at 4°C. DNA was stained with ethidium bromide and viewed on a UV transilluminator. Full gel images are shown in Supplementary Figure S1.

### ChIP-seq

Experiments were done according to the protocol of Haycocks *et al.* (22) using strain RPB104Δ*hns* that encodes SPA-tagged *rpoS*. Duplicate cultures were grown to an OD_600_ of 3.0 in LB then crosslinked, lysed and sonicated. Next, σ^38^-DNA complexes were immunoprecipitated with anti-FLAG antibody and Protein A sepharose beads. Fragments were blunted and poly(A) tailed with 5′-3′ exo- Klenow (NEB). NEXTflex barcoded adaptors (Bioo Scientific) were attached by ligation. Following elution, complexes were de-crosslinked by boiling. The resulting DNA libraries were amplified by PCR and quantified by Qubit analysis before pooling and sequencing using an Illumina MiSeq instrument. The raw data are available from ArrayExpress (E-MTAB-8778).

### Bioinformatics

FastQ files were converted to Sanger format using FastqGroomer and aligned to the MG1655 genome using Bowtie for Illumina. The resulting SAM files were converted to BAM files and read depth per base was calculated using MultiBam summary. Data were normalised to the same average read count to allow comparison. Peaks for σ^38^ binding were called if the average read depth was 4 or above. The same analysis was applied to results of a ChIP-seq assay of σ^38^ binding in the parent strain RPB104 (23). To identify motifs in collections of putative promoter DNA sequences we used MEME (24).

## RESULTS

### Happenstance promoters share a conserved AT-rich sequence element

**Figure 1.**
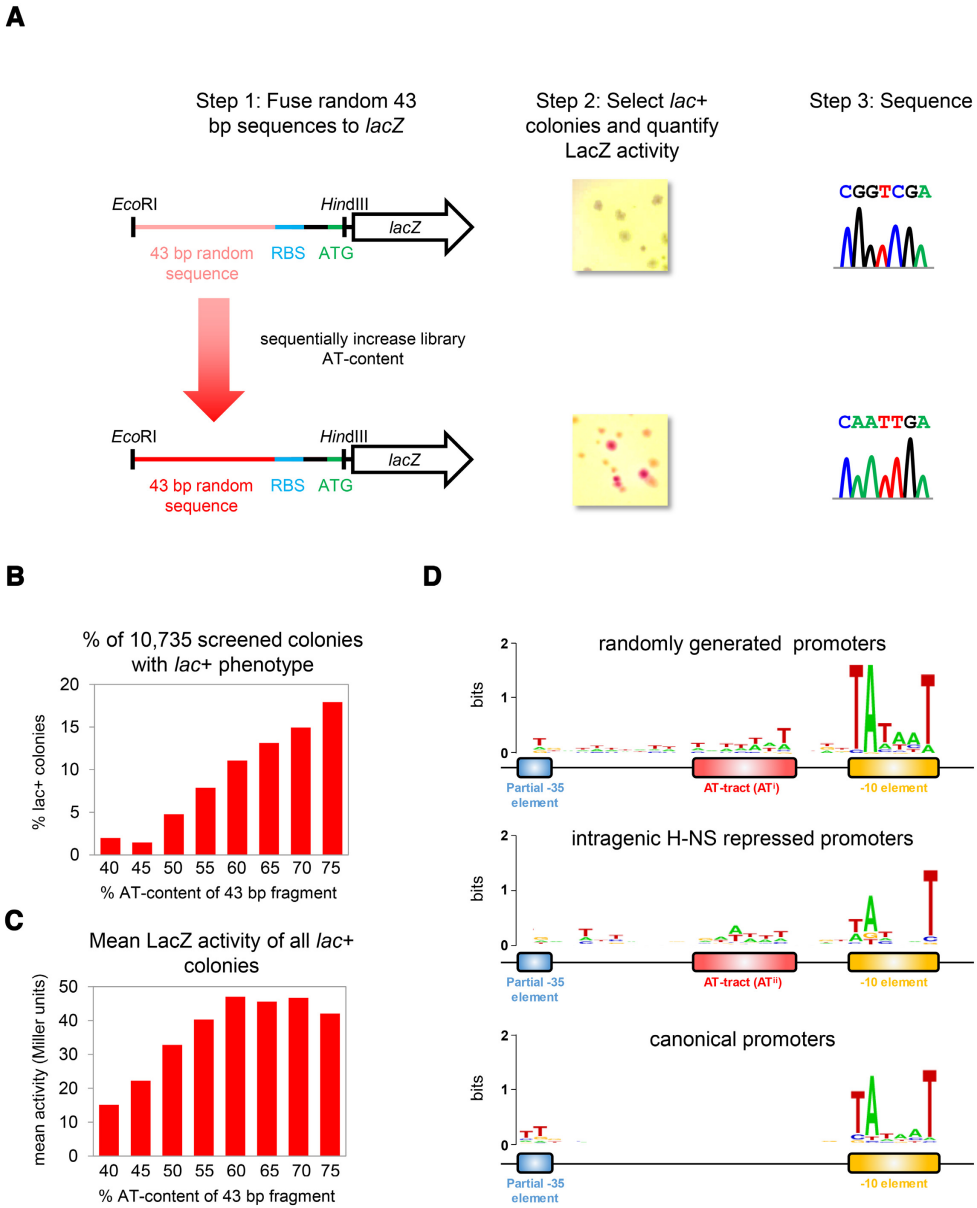
Promoters are more prevalent and active in random DNA sequences with a higher AT-content. (**A**) Experimental strategy for generation and selection of promoters from random DNA sequences of defined AT-content. Briefly, 43 bp DNA fragments of random sequence, but a defined AT-content between 40% and 75%, were fused to the *lacZ* gene in plasmid pRW50. The resulting plasmid libraries were used to transform the *E. coli* Δ*lac* strain JCB387. Transformants expressing LacZ were identified as red or pink colonies on MacConkey agar. Such *lac+* colonies were selected and their random 43 bp DNA insertion was sequenced. (**B**) The number of *lac*+ colonies increases as AT-content increases. A total of 10,735 colonies were examined and the percentage of *lac+* colonies is plotted against the % AT-content of the corresponding insertion library. (**C**) Average promoter activity increases in AT-rich DNA sequences. The LacZ activity, quantified for *lac+* colonies by β-galactosidase assay, is plotted against library % AT-content. (**D**) DNA sequence motifs associated with different classes of promoter

The starting point for this work was our previous analysis of promoters within horizontally acquired genes (8,10). We speculated that such promoters were chance occurrences resulting from the high AT-content of foreign DNA (10). To quantify the relationship between DNA AT-content and promoter occurrence we generated 8 separate DNA fragment libraries. The fragments in each library were 43 bp in length and had random sequences. However, the overall AT-content of libraries was different and set between 40% and 75%. Fragments were fused to *lacZ* in plasmid pRW50 and used to transform *E. coli* strain JCB387. A total of 10,735 transformants were selected on MacConkey agar. This allowed 1,039 red *lac*+ colonies to be identified, corresponding to active promoters. Our experimental strategy is summarised in Figure 1A. For each fragment library, we calculated the percentage of all DNA fragments with promoter activity. These data are plotted against percentage AT-content in Figure 1B. There was a clear correlation between library AT-content and the number of promoters identified. Few promoters were generated in DNA fragments with an AT-content <50%. We also measured LacZ activity in lysates of cultures derived from each *lac*+ colony. This allowed us to determine the average activity of all promoters in each library (Figure 1C). Whilst a positive correlation was evident, there was no increase in average promoter activity when the AT-content exceeded 60% (Figure 1C). Each active promoter was sequenced and a DNA sequence logo was generated (Figure 1D, top). For comparison, we also made DNA sequence logos representing intragenic promoters subject to repression by H-NS (middle) and canonical intergenic promoters (bottom) (10,25). In all logos, the best conserved feature was the promoter -10 element, particularly bases one, two and six. Conversely, -35 elements were poorly conserved; only the 5′-TT-3′ dinucleotide at positions one and two was evident. Randomly generated and intragenic promoters had an AT-tract between promoter positions -17 and -23. This element was not enriched at canonical promoters. Note that AT-tract sequence differed in randomly generated and intragenic promoters (Figure 1D, compare top two panels). We subsequently refer to these variants as AT^i^ and AT^ii^.

### AT-tracts can activate cryptic -10 elements

**Figure 2.**
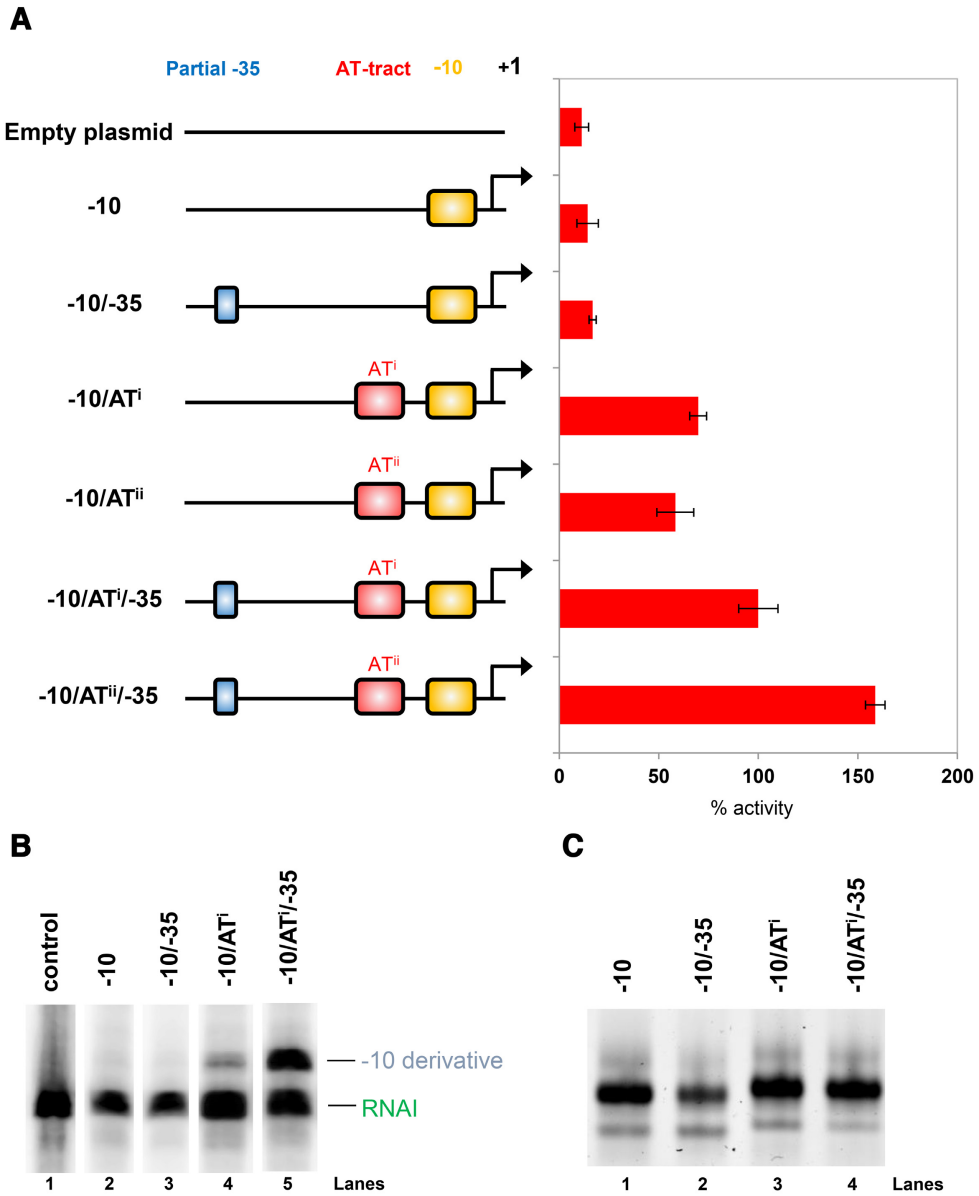
AT-tracts activate transcription from cryptic -10 elements and alter DNA bending. (**A**) AT-tracts increase promoter activity. Synthetic promoter sequences containing a consensus -10 element (yellow box), -35 element (blue box) and two different AT-tract sequences (AT^i^ and AT^ii^, red box) were cloned upstream of the *lacZ* gene. For each promoter, LacZ activity was measured in triplicate. Activity values are shown as a percentage relative to the ‘-10/AT^i^/-35’ promoter. The absolute activity of this promoter was 236 Miller units. Error bars show standard deviation. (**B**) An AT-tract is required for transcription initiation *in vitro*. Bands on the gel are RNA transcripts produced *in vitro* using the indicated promoter sequences. The RNAI transcript serves as an internal control. The control lane shows transcripts generated from empty pSR plasmid. (**C**) AT-tracts alter DNA bending. Bands on the gel correspond to DNA fragments with or without an AT-tract. All DNA fragments are the same length but have different electrophoretic mobility due to altered curvature.

As noted above, the promoter -10 element alone is ineffective. Hence, transcription factors play a key role by activating canonical promoters. We reasoned that the AT-tract may circumvent the need for transcription factors, or a promoter -35 element, at happenstance promoters. To test this, we generated a set of synthetic promoters. The promoters all had a consensus -10 element. This was augmented with combinations of AT^i^, AT^ii^ and a partial -35 hexamer (5′-TT-3′). The different promoters are illustrated schematically alongside LacZ activity measurements in Figure 2A. As expected, the -10 element alone was unable to drive *lacZ* expression. Addition of the partial -35 element had no impact. Conversely, addition of either AT-tract variant resurrected promoter activity. This activation increased further when a partial -35 element was also present. To confirm our observations, selected promoters were cloned upstream of the *λoop* terminator in plasmid pSR. Transcripts terminating at *λoop* can be detected following electrophoresis. The RNAI transcript is derived from the pSR replication origin and serves as an internal control. No transcripts were produced from promoters lacking AT-tracts (Figure 2B, lanes 1–3). Addition of an AT-tract allowed transcription (lane 4) that increased further upon inclusion of the partial -35 element (lane 5).

### AT-tracts alter promoter DNA bending

Changes to the DNA sequence, particularly the introduction of AT-tracts, can alter DNA bending (17). Altered bending affects electrophoretic mobility of DNA during native PAGE. We examined the mobility of different promoters with or without an AT-tract (Figure 2C). DNA fragments containing a -10 element, with or without a partial -35 hexamer, had the same electrophoretic mobility (Figure 2C, lanes 1 and 2). Addition of an AT-tract reduced mobility of DNA fragments during electrophoresis (lanes 3 and 4).

### Most AT-tract sequences stimulate transcription

**Figure 3.**
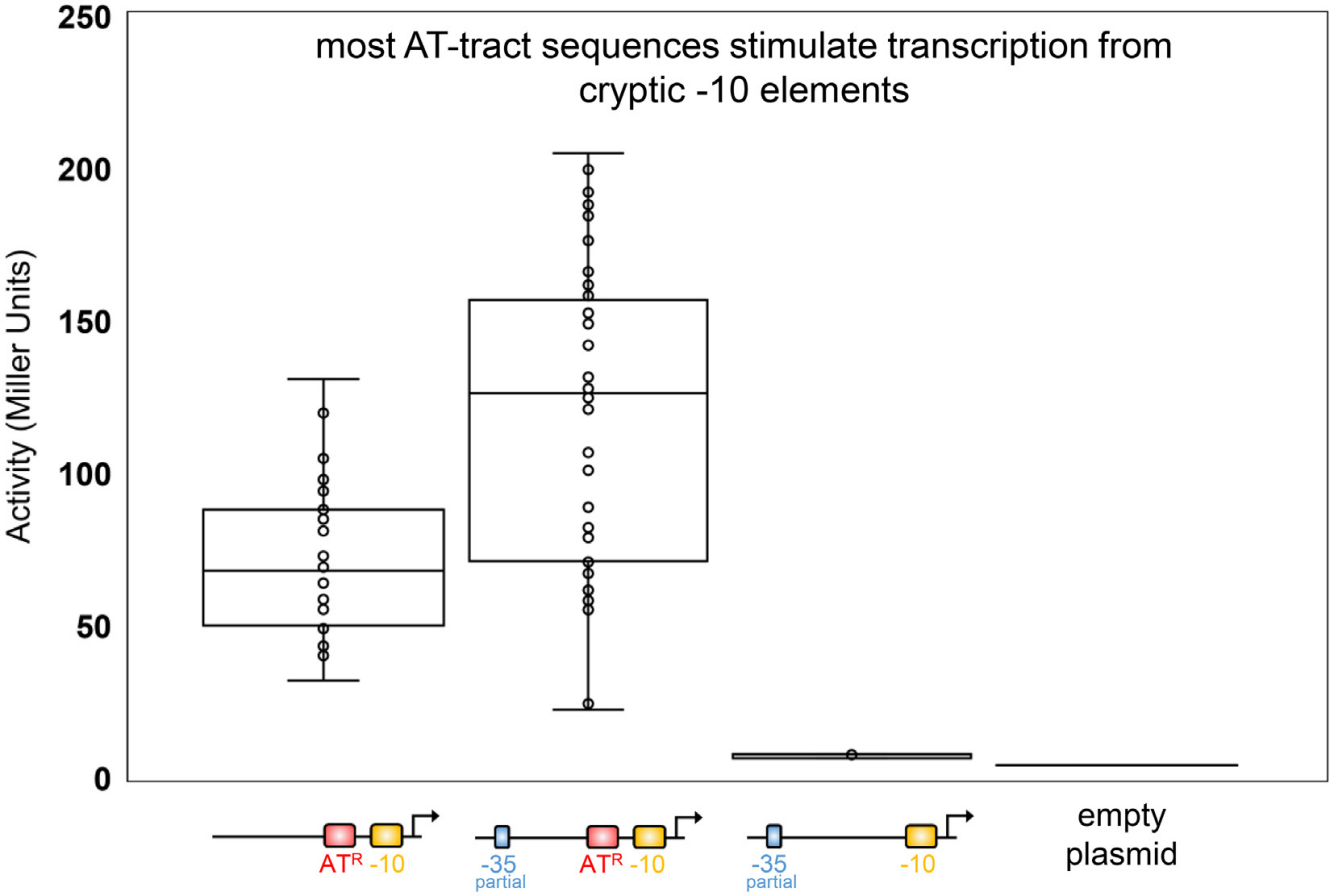
AT-tracts of any sequence can activate transcription from a cryptic -10 element. Promoter sequences containing a -10 element and a randomised AT-tract, with or without a partial -35 element, were fused to the *lacZ* gene in plasmid pRW50. The resulting library was used to transform the Δ*lacE. coli* strain JCB387 and LacZ activity was determined for each transformant. Data are presented as a box plot with each point representing one colony. Measured LacZ activity is also shown for a promoter without an AT-tract (-10/-35) and the empty plasmid.

Interestingly, both AT^i^ and AT^ii^ were able to activate transcription despite having different sequences (Figure 2A). We reasoned that many AT-tracts may be able to stimulate transcription. To test this we made a new library of promoter DNA fragments. The fragments had a consensus -10 hexamer in the presence or absence of a partial -35 element. Promoter positions -17 to -23 were a random combination of As and Ts (denoted AT^R^). We examined 103 promoters containing AT^R^; all were transcriptionally active (Figure 3). This was evident both in the presence (44 promoters) and absence (59 promoters) of the partial -35 element. We conclude that most, and potentially all, appropriately positioned AT-tracts stimulate transcription. In turn, this suggests that a broad range of DNA conformations can be beneficial.

### Activation by AT-tracts requires σ^70^ residue R451

**Figure 4.**
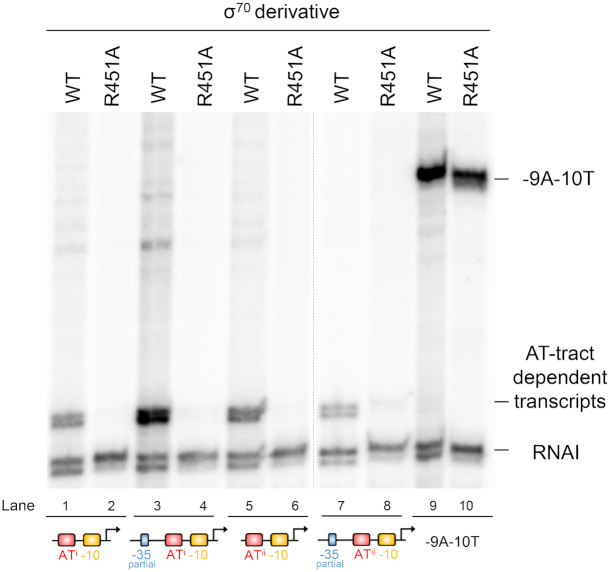
Sidechain R451 of σ^70^ is required for transcription at promoters dependent on an AT-tract. (**A**) The gel image shows transcripts generated by RNA polymerase associated with either WT σ^70^ or the R451A mutant. The RNAI transcript is derived from the pSR replication origin and serves as an internal control. The -9A-10T promoter is constitutively active and does not have an AT-tract.

We previously showed that an A or T at promoter position -18 could stimulate transcription by enhancing a DNA backbone contact with σ^70^ side chain R451 (17). We predicted that σ^70^ R451 would also be important at promoters dependent on the AT-tract. To test this, we repeated our *in vitro* transcription analysis and compared wild type RNA polymerase with the σ^70^ R451A derivative. The R451A mutation resulted in a total loss of transcription at all promoters dependent on an AT-tract (Figure 4, lanes evenly numbered up to 8). However, the mutant σ^70^ was unimpaired at a control promoter (lanes 9 and 10). The control promoter is dependent on a near consensus -35 hexamer and has a G at position -18 (17).

### Many H-NS repressed intragenic promoters require AT-tracts

**Figure 5.**
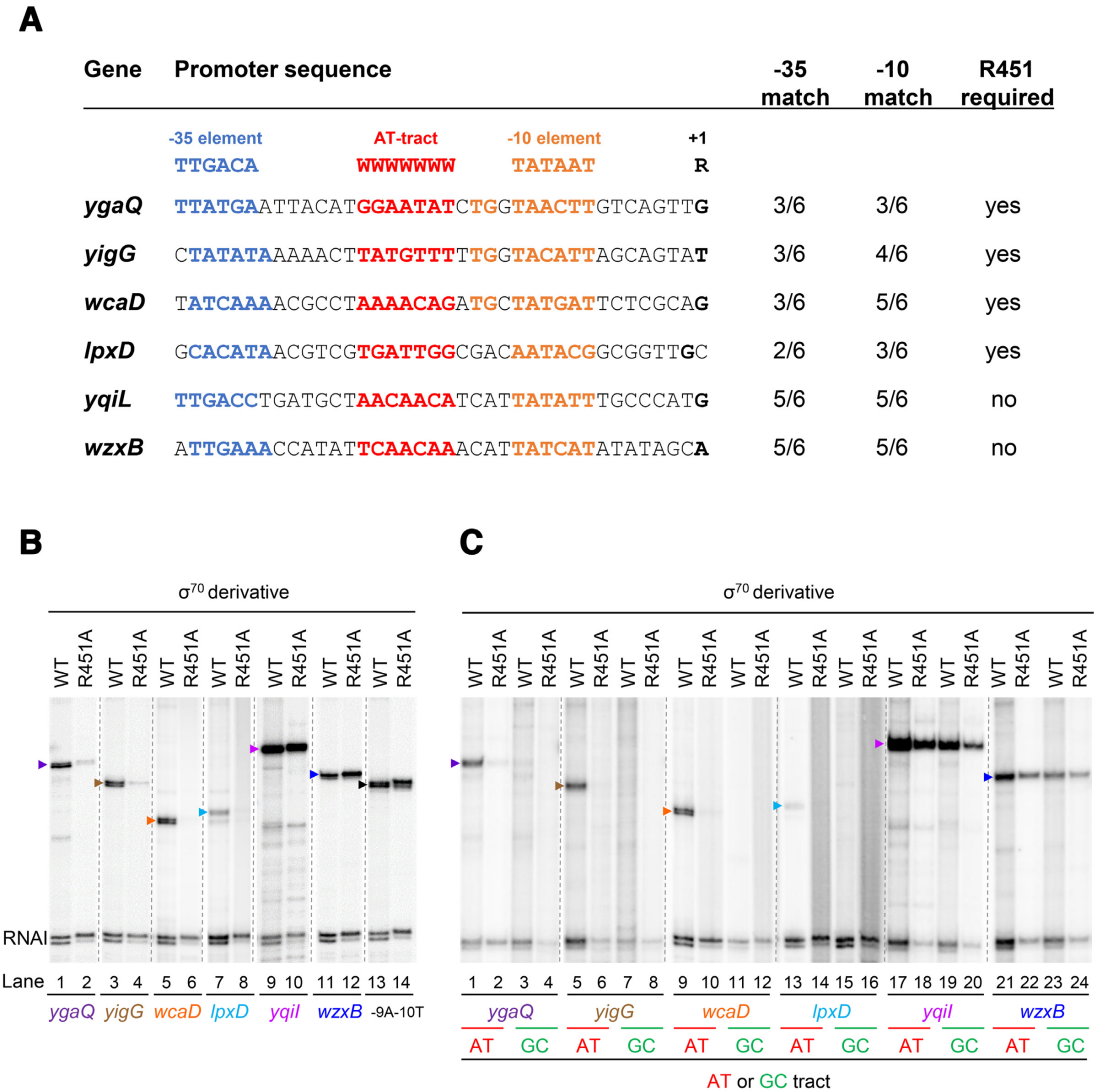
Effect of the σ^70^ R451A mutation on spurious intragenic promoters. (**A**) Intragenic promoter DNA sequences. Transcription start sites and promoter elements are highlighted. The number of base matches to the consensus sequence for each element is also indicated. (**B**) The gel image shows transcripts generated by RNA polymerase associated with either WT σ^70^ or the R451A mutant. Bands at 107/108 nt are RNAI transcripts derived from the pSR replication origin. The -9A-10T is constitutively active and functions independently of the AT-tract. (**C**) Effect of replacing AT-tracts with GC-rich sequences (the -35 and -10 elements were unchanged). The sequence immediately upstream of the -10 element was replaced with 5′-CTTCGCAGTCAGCTGAC-3′ (or 5′-CTTCGCAGTCAGCT-3′ for extended -10 elements).

We speculated that many naturally occurring promoters, within H-NS silenced genes, would be dependent on AT-tracts and σ^70^ R451 for activity. To test this prediction, we used six intragenic promoters from our previous analysis of horizontally acquired genes (10,26). The promoters were within the coding sequences of *ygaQ, yigG, wcaD, lpxD, yqiI* or *wzxB*. We determined the size of transcripts generated from each promoter *in vitro* (Supplementary Figure S2). This allowed transcription start sites to be mapped. The annotated promoter sequences are shown in Figure 5A. All six promoters contain an appropriately positioned AT-rich sequence. However, only the promoters within *yqiI* and *wzxB* had -35 and -10 sequences near to the consensus. Transcription was measured *in vitro* using RNA polymerase or the σ^70^ R451A derivative. Production of the *ygaQ, yigG*, *wcaD* and *lpxD* derived transcripts was greatly reduced by the R451A mutation (Figure 5B, lanes 1–8). Conversely, transcription from the *yqiI* and *wzxB* DNA fragments was unchanged (lanes 9–14). We next replaced the AT-tract upstream of each -10 element with a GC-rich sequence. Only promoters requiring σ^70^ R451 were inactivated when the AT-tract was removed (Figure 5C, lanes 1 and 3, 5 and 7, 9 and 11, 13 and 15). Conversely, promoters not requiring R451 functioned independently of the AT-tract (lanes 17–24).

### Many H-NS repressed intragenic promoters function with σ^38^ associated RNA polymerase

**Figure 6.**
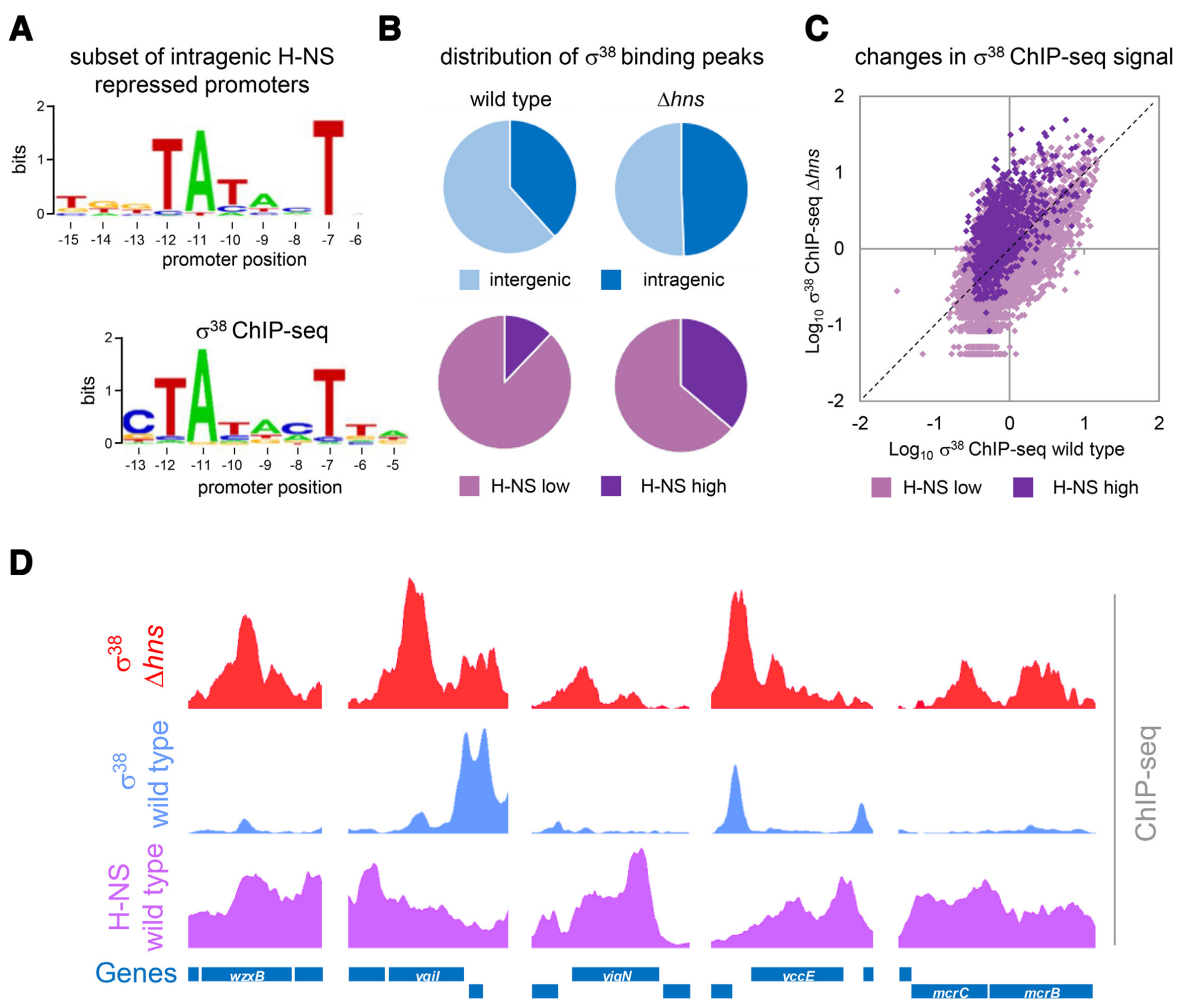
The alternative σ^38^ factor recognises many intragenic promoters. (**A**) Sequence logo showing the imperfect extended -10 element conserved in randomly generated promoters (top). The σ^38^ promoter logo defined using ChIP-seq analysis is included for comparison (23). (**B**) The proportions of σ^38^ ChIP-seq peaks found inside/outside of genes (blue charts) or regions highly H-NS bound by H-NS (purple charts) in a wild-type (WT) or Δ*hns E. coli* strain. (**C**) Examples of σ^38^ ChIP-seq peaks inside genes in Δ*hns* (red) but not WT (blue) cells. H-NS binding signals are derived from the ChIP-seq data of Kahramanoglou *et al.* (32). (**D**) Scatter plot showing changes in σ^38^ binding when *hns* is deleted. Each data point represents the log_10_ of σ^38^ ChIP-seq signal in a 500 bp bin. Sequences usually bound by H-NS are shown in dark purple (high H-NS) and areas without H-NS are shown in pale purple (low H-NS).

Our search for promoter motifs provided evidence that AT-tracts play a key role at happenstance promoters (Figure 1D). Hence, we have focused on understanding this DNA element and its interaction with RNA polymerase. However, during our initial promoter motif analysis, we identified a second DNA logo associated with horizontally acquired genes (Figure 6A, top). The logo depicts a 5′-TGn-3′ motif upstream of the sequence 5′-TATACT-3′. Previous work has shown that promoters used by the alternative σ^38^ factor, encoded by *rpoS*, often have 5′-TGn-3′ motifs (27). Furthermore, ChIP-seq analysis identified 5′-TATACT-3′ as the consensus -10 element for σ^38^ (Figure 6D, bottom) (23). Hence, σ^38^ might also serve promoters within horizontally acquired genes. To test this, we used ChIP-seq and compared chromosome-wide σ^38^ binding in *E. coli* RPB104 and the *Δhns* derivative. We identified 890 σ^38^ binding peaks in the starting strain and 905 peaks in the *Δhns* derivative (Supplementary Table S1). The proportion of σ^38^ binding peaks within genes increased in cells lacking H-NS (Figure 6B, blue charts). Similarly, there was increased binding of σ^38^ in H-NS targeted regions (labelled H-NS high) if H-NS was absent (Figure 6B, purple charts). We previously showed that deleting *hns* reorganised global RNA polymerase positioning; binding increased at H-NS silenced genes but was reduced at most other loci due to titration of the limited RNA polymerase pool (8). To understand if this was also the case for σ^38^ we divided the genome into 500 bp sections. We then calculated the σ^38^ binding signal for each DNA segment in wild type and Δ*hns* cells. The values are plotted in Figure 6C. The diagonal line indicates where data points fall if the σ^38^ binding signal is the same in each strain. In the Δ*hns* strain, σ^38^ binding signals increased at regions formerly bound by H-NS (H-NS high). Conversely, σ^38^ binding decreased at most other genomic regions (H-NS low). Figure 6D illustrates ChIP-seq peaks for σ^38^ binding within H-NS targeted genes. Multiple peaks for intragenic σ^38^ binding are evident in Δ*hns* (red) but not wild type (blue) cells.

## DISCUSSION

We previously noted the widespread spurious transcription of AT-rich horizontally acquired genes (10). The phenomenon was attributed to increased occurrence of sequences resembling promoter -10 elements (8,10). However, promoter -10 elements alone are unable to drive transcription; the sequence cannot recruit RNA polymerase to the DNA (4). Structural analysis provides a rationale for this observation; base specific interactions between σ^70^ and the -10 hexamer only occur after DNA unwinding (5). In this work we show that spurious intragenic promoters frequently depend on an AT-tract located between -17 and -23 base pairs upstream of the transcription start site (Figures 2–5). Consistent with this, we and others have previously noted an A or T at positions -17 and -18 can be stimulatory (17,28–30). The AT-tract alters nucleic acid bending and facilitates a contact between σ^70^ side chain R451 and the double helix backbone. Hence, otherwise cryptic -10 elements are able to participate in the process of transcription initiation. Many intragenic promoters also function with σ^38^ bound RNA polymerase. This may explain why inactivation of *rpoS* is a pre-requisite for deletion of *hns* in *Salmonella* spp. (31).

The simplest explanation for the abundance of promoters within AT-rich genes is chance occurrence (8–10). However, this hypothesis is difficult to test. In an effort to address the issue, we compared DNA sequence properties of spurious and randomly generated promoters (Figure 1). Whilst not proof of accidental origin, both types of promoter frequently depend on the presence of an AT-tract. Furthermore, the AT-tract is not enriched at canonical promoters. Presumably, this is to avoid constitutive promoter activity that short circuits regulation by transcription factors. We suggest that the AT-tract occurs frequently because of its low information content; any AT-rich DNA sequence is stimulatory (Figure 3). Conversely, promoter -35 elements have a precise consensus that is less likely to arise spontaneously.

Intriguingly, the frequency of chance promoter occurrence increases only when the DNA AT-content exceeds that of the *E. coli* genome (i.e. ∼50%, Figure 1B). This may be indicative of adaptations that reduce the initiation of transcription at unwanted locations. For instance, the *E. coli* RNA polymerase could be hardwired to initiate transcription inefficiently at DNA sequences with an AT-content resembling coding DNA. We speculate that the AT-content threshold, above which promoters spontaneously arise, will differ depending on the genome AT-content of a given organism. Similarly, we predict that RNA polymerases isolated from bacteria with AT-rich genomes could be adapted to such templates and generate fewer spurious transcripts compared to the *E. coli* enzyme. In summary, we provide an explanation for the widespread occurrence of spurious promoters within horizontally acquired sections of the *E. coli* genome. Our data also have implications for our understanding of RNA polymerase specificity and promoter evolution.

## DATA AVAILABILITY

ArrayExpress accession E-MTAB-8778.

## Supplementary Material

gkaa244_Supplemental_Files

## Notes

Present address: Shivani S. Singh, MRC London Institute of Medical Sciences, London, UK.

Present address: Alicia G. Gubieda, Institute for Cell and Molecular Biosciences, Newcastle University, Newcastle upon Tyne NE2 4HH, UK.
