# Supplementary material for: A non-canonical promoter element drives spurious transcription of horizontally acquired bacterial genes
Source: Nucleic Acids Res. 2020 Apr 16;48(9):4891–901. doi: 10.1093/nar/gkaa244 (PMC7229825; doi:10.1093/nar/gkaa244)
Supplement: gkaa244_Supplemental_Files [file gkaa244_supplemental_files.zip › Warman_et_al_2_supplementary_figures_300320.pdf]

Figure S1

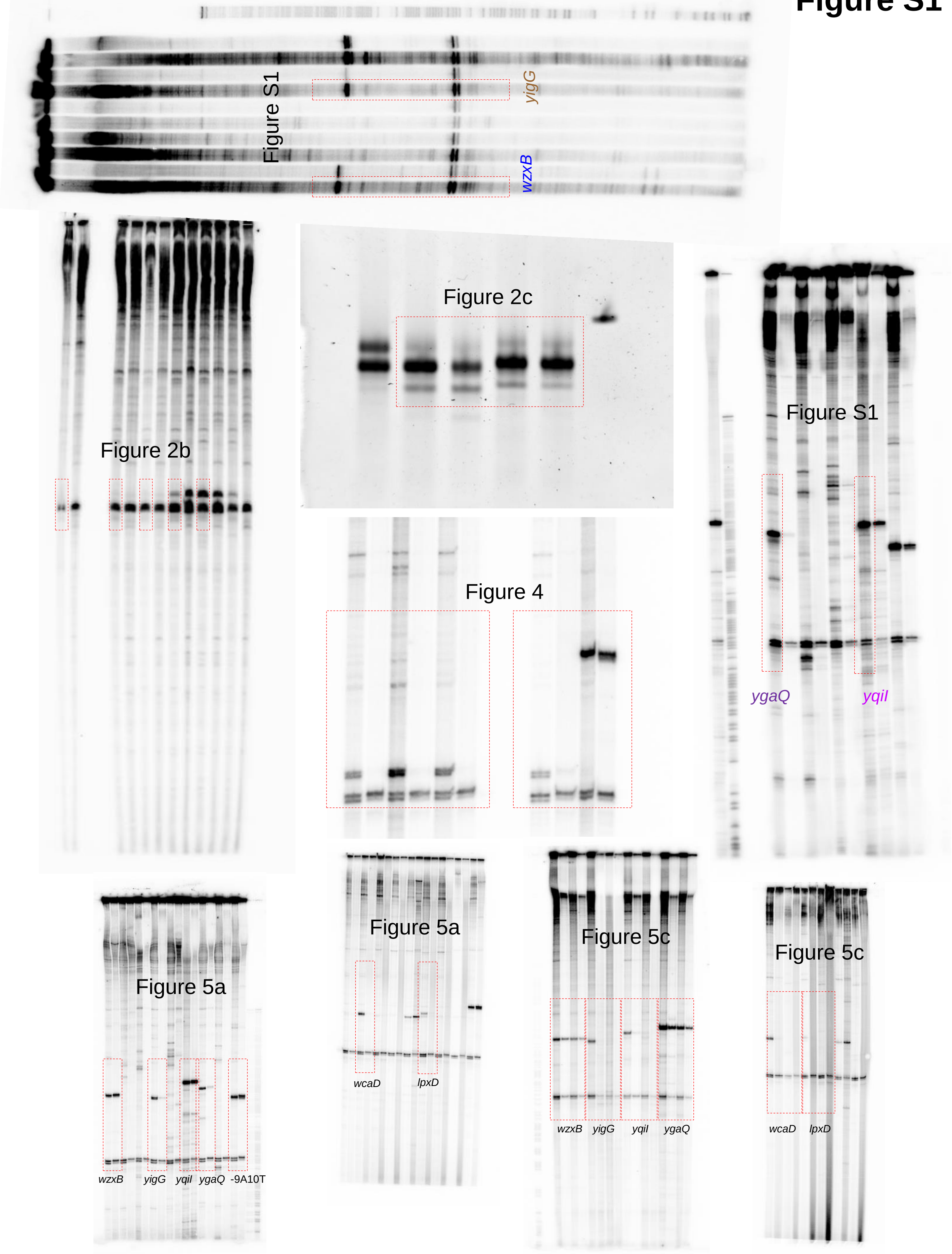

Figure S1
yigG
wzxB
Figure 2b
Figure 2c
Figure S1
ygaQ
yqiI
Figure 4
Figure 5a
wzxB
yigG
yqiI
ygaQ
-9A10T
Figure 5a
wcaD
lpxD
Figure 5c
wzxB
yigG
yqiI
ygaQ
Figure 5c
wcaD
lpxD

Figure S1: Original gel images. The figure shows full uncropped gel images used elsewhere in the manuscript. The portions of gels used, and the figure to which they correspond, are boxed and labelled respectively.

Figure S2

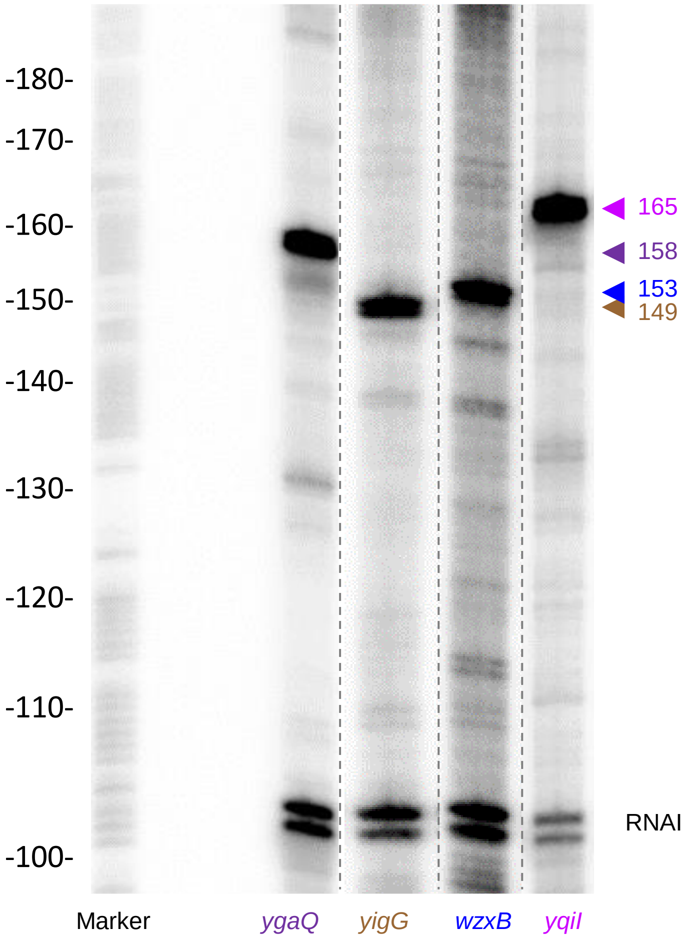

Figure S2: Transcript size determination. The image shows result of *in vitro* transcription assays. Key transcripts are indicated and an arbitrary G+A sequence ladder was also analysed for calibration. Once transcript lengths were defined transcription start sites could be identified by counting back the requisite number of nucleotides from the loop transcriptional terminator in plasmid pSR, the template for intro transcription carrying cloned promoters.
